# Supplementary material for: A scoping review of patient engagement activities during COVID-19: More consultation, less partnership
Source: PLoS One. 2021 Sep 29;16(9):e0257880. doi: 10.1371/journal.pone.0257880 (PMC8480845; doi:10.1371/journal.pone.0257880)
Supplement: S1 Table — (DOCX) [file pone.0257880.s001.docx]

S1 Table. MEDLINE search strategy

| **#** | **Search Term** | **Results** |
| --- | --- | --- |
| 1 | Exp coronavirus infections/ OR exp coronavirus/ | 18,460 |
| 2 | (coronavirus* OR corona-virus* OR coronovirus* OR corono-virus* OR 2019-nCoV OR COVID OR COVID-19 OR CORVID-19 OR WN-CoV OR HcOv-19 OR 2019 novel OR Ncov OR SARS-CoV-2 OR SARS-CoV2 OR SARSCoV-2 OR SARSCoV2 OR SARSCov19 OR SARS-CoV19 OR SARS-CoV-19 OR (Wuhan AND coronavirus)).tw,kw,kf. | 27,369 |
| 3 | 1 OR 2 | 34,837 |
| 4 | Patient participation/ OR consumer participation/ OR patient advocacy/ OR consumer advocacy/ | 67,958 |
| 5 | (Patient* OR famil* OR consumer* OR care?giver* OR carer* OR user* OR client* OR visitor*) ADJ3 (engag* OR participat* OR involv*OR consult* OR design* OR deliver* OR develop* OR plan* OR evaluat* OR advoca* OR empower* OR activat* OR partner* OR advisor* OR committee* OR experienc*).tw,kw,kf. | 692,918 |
| 6 | 4 OR 5 | 750,469 |
| 7 | 3 AND 6 | 578 |
| 8 | Limit to 2019-2020 | 395 |
