# Supplementary material for: A scoping review of patient engagement activities during COVID-19: More consultation, less partnership
Source: PLoS One. 2021 Sep 29;16(9):e0257880. doi: 10.1371/journal.pone.0257880 (PMC8480845; doi:10.1371/journal.pone.0257880)
Supplement: Attachment — Submitted filename: Response to Reviewers.docx [file pone.0257880.s004.docx]

July 29, 2021

Mariusz Duplaga

Academic Editor

PLOS ONE

**RE: Response to Reviews**

Manuscript Title: A scoping review of patient engagement activities during COVID-19: More consultation than partnership (PONE-D-20-38436R1)

Dear Dr. Mariusz Duplaga,

Thank you for considering and seeing potential in our manuscript. We appreciate the reviewers’ comments and have outlined a detailed response to each of the comments, along with a reference to the page number where the changes can be found in the main paper (track changes version).

| **Journal Requirements** | **Author Responses** |
| --- | --- |
| Please review your reference list to ensure that it is complete and correct. If you have cited papers that have been retracted, please include the rationale for doing so in the manuscript text, or remove these references and replace them with relevant current references. Any changes to the reference list should be mentioned in the rebuttal letter that accompanies your revised manuscript. If you need to cite a retracted article, indicate the article’s retracted status in the References list and also include a citation and full reference for the retraction notice. | We have reviewed the reference list to ensure that it is complete and correct. |
| **Reviewer 2** | **Author Responses** |
| Thank you to the authors for reflecting on my feedback and revising the manuscript. I have only a few outstanding comments.  On further reflection, I realized that nowhere in the manuscript is the concept of engagement defined. Adding a definition in the introduction would increase clarity for readers. Also, could the authors please explain in the methods how engagement was determined—did papers self-identify as engagement and use the terminology, or did the reviewers identify engagement based on a comparison of their definition to reported descriptions in the included manuscripts? These points have implications for the interpretation of the findings, particularly in to the more superficial types of engagement in patient care noted by the authors. | Thank you for noting this. Engagement was described, but we have re-integrated the definition in the introduction to increase clarity for the readers.  Please refer to the Study Selection section of the methods for the explanation of how engagement was determined. The described activity had to align with at least one of the three core levels of engagement (along the continuum) as outlined in Carman and colleagues’ framework for patient and family engagement. |
| I struggled with the statements in the study selection section around context. given publishing timelines, would it not be more relevant to clarify if the engagement activities occurred during the pandemic? Presumably would many studies published in early 2020 actually be reporting on issues/ activities from before the pandemic? I’m unclear how this was determined. | Please refer to our first inclusion criteria in the Study Selection section of the methods. Articles were required to be specific to COVID-19. So, while it is possible for studies published in early 2020 to be reporting on activities from before the pandemic, for this review, we required the articles to be specific to the context of the pandemic. If articles did not clearly state that the engagement activity was occurring during the pandemic, then it was excluded. |
| The organization and headings in table 2 are confusing to me. “Challenges” and “Lessons Learned” are not characteristics of strategies. Also, “Engagement category” column is reporting sub-categories. All told this table doesn’t align with the text reporting of results and is difficult to follow. | We have changed the title of Table 2 to ‘Characteristics of patient engagement activities’ to reduce potential confusion around the challenges and lessons learned columns. These columns are specific to the context of the activities and provide important contextual information. We have revised the “Engagement Category” column heading to reflect what is reported. We have reorganized the table to better align with the text presentation of the results. |
| The revisions to the reporting of the findings through categories was generally helpful. However on further reflection, I question whether the sub-category “technology for sharing information” should be categorized as engagement, as this really seems to me to better align with classic definitions of dissemination of information and one way transmission of communication. Based on the descriptions provided, there is no evidence of any interaction, therefore I’m not sure it should be called engagement. This is also why adding definitions to clarify what the authors mean by engagement would be helpful here. | We appreciate this comment and acknowledge that the use of technology for sharing information may be more of a one way transmission of communication. However, based on Carman and colleagues’ framework for patient and family engagement that was used to categorize engagement activities in this scoping review, these types of activities occur along the continuum of engagement and thus, we chose to include them. Please refer to the introduction for the definition of patient engagement, as well as an explanation of the framework used for conceptualizing and categorizing the engagement activities. |
| The statement on line 78-81, “While these examples refer to more ‘active’ forms of engagement, activities can be more broad in nature, ranging -- from clinical consultations to partnership activities (advisory groups and participation in policy decision making)” is important and requires a reference. | We have added a reference to this statement. |
| There are some incorrect references that need to be cleaned up (e.g. 21). | We have corrected the one invalid reference. |

The reviewers’ insights strengthened our paper and we hope that we have addressed all comments to your satisfaction.
